# Knee Arthrodesis Using the Masquelet Technique for Postoperative Uncontrolled Infection and Extensor Mechanism-Deficient Total Knee Arthroplasty

**DOI:** 10.7759/cureus.86315

**Published:** 2025-06-18

**Authors:** Kengo Abe, Minoru Takasaki, Shingo Fukagawa

**Affiliations:** 1 Department of Orthopaedic Surgery, National Health Organization (NHO) Kokura Medical Center, Kitakyushu, JPN; 2 Department of Orthopaedic Surgery, Kyushu Rosai Hospital, Kitakyushu, JPN

**Keywords:** infection, knee, knee arthrodesis, masquelet technique, total knee arthroplasty (tka)

## Abstract

Total knee arthroplasty (TKA) is a widely performed surgical procedure known for its favorable long-term outcomes. However, postoperative infection poses significant treatment challenges and may lead to severe complications. This case involves a 55-year-old woman who developed a patellar fracture following TKA, complicated by deep infection after osteosynthesis and accompanied by loss of extensor mechanism. Despite undergoing multiple surgeries, the treatment process proved difficult. Ultimately, knee arthrodesis with bilateral plates was performed using the Masquelet technique. The postoperative course was favorable, with successful bone union achieved. Although the patient experienced a leg length discrepancy, she regained the ability to walk short distances. Knee arthrodesis employing the Masquelet technique proved effective for infection control, bone regeneration, and management of extensor mechanism deficiency, making it a viable treatment option for managing postoperative infections after TKA.

## Introduction

Total Knee Arthroplasty (TKA) is a common surgical procedure known for its favorable long-term postoperative outcomes. In Japan, TKA is widely performed, and the number of procedures is expected to increase over the next decade [1,2]. However, postoperative infection can present significant treatment challenges and lead to serious complications.

We present a case in which a patellar fracture occurred following TKA. After osteosynthesis, the patient developed a refractory deep infection, which led to redisplacement of the fracture and necessitated patellectomy, ultimately resulting in loss of extension function and necessitating knee joint arthrodesis. In this case, the Masquelet technique [3], a well-established method for treating infected nonunion, was applied to the knee joint, achieving favorable clinical outcomes.

To the best of our knowledge, there have been no previous reports of knee joint arthrodesis using the Masquelet technique. Therefore, we report this case to contribute to the literature on the management of complex post-TKA complications.

## Case presentation

A 55-year-old woman, height 153 cm, weight 68 kg, BMI 29.1 kg/m², with bipolar disorder, who was able to live independently, presented with severe right knee pain that had progressively worsened over the past two years, rendering her unable to walk. She was referred to our institution by a local physician. Given her desire to regain mobility and perform activities of daily living (ADL), TKA was planned.

Due to her psychiatric condition, she was on multiple psychotropic medications, which resulted in significant cognitive impairment. Consequently, her hospitalization for the surgical procedure was arranged in a psychiatric ward to ensure appropriate management of her mental health.

**Figure 1:**
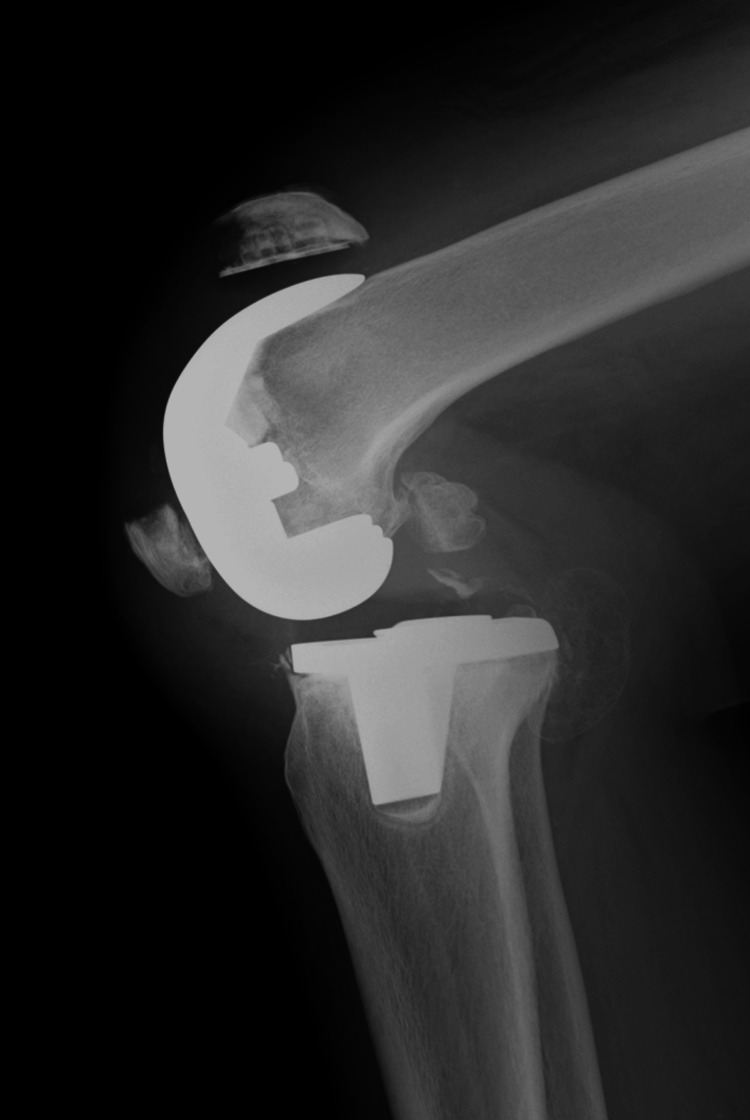
Injury and before initial surgery X-ray imaging at the time of injury revealed a right patellar fracture.

She underwent primary TKA. Although the degeneration of the patellofemoral joint was mild, our institution routinely replaces the patella in all cases. Accordingly, the patella was replaced as per standard protocol. Postoperatively, she underwent rehabilitation and was discharged home after recovering to an ADL level sufficient for independent living. Approximately three months after surgery, she fell at home and struck her right knee. It is presumed that she sustained a patellar fracture at that time; however, as she was able to crawl around inside the house, she did not seek medical attention. A few weeks later, her pain worsened, and she was transported to the emergency department of our hospital. After transport, her X-ray revealed a right patellar fracture (Figure 1).

**Figure 2:**
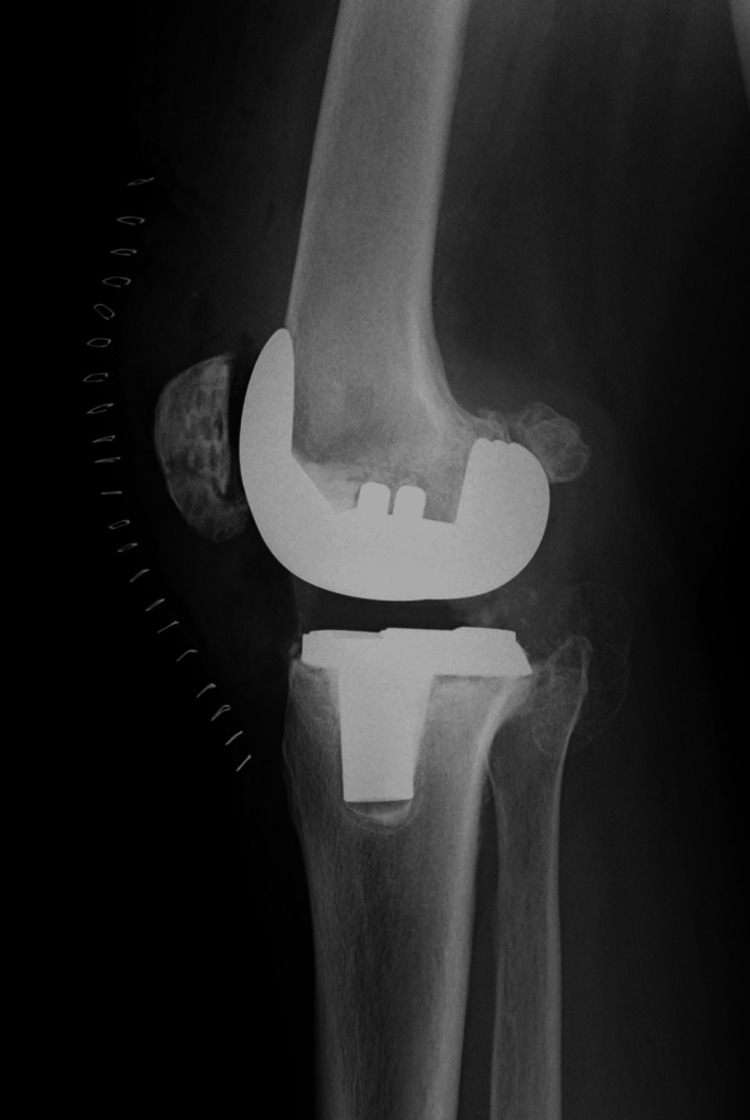
Postoperative X-ray after the initial surgery Osteosynthesis was performed with a Nesplon cable (Alfresa Pharma Corporation, Osaka, Japan). No loosening of the patellar component was observed during the operation and postoperative X-ray.

**Figure 3:**
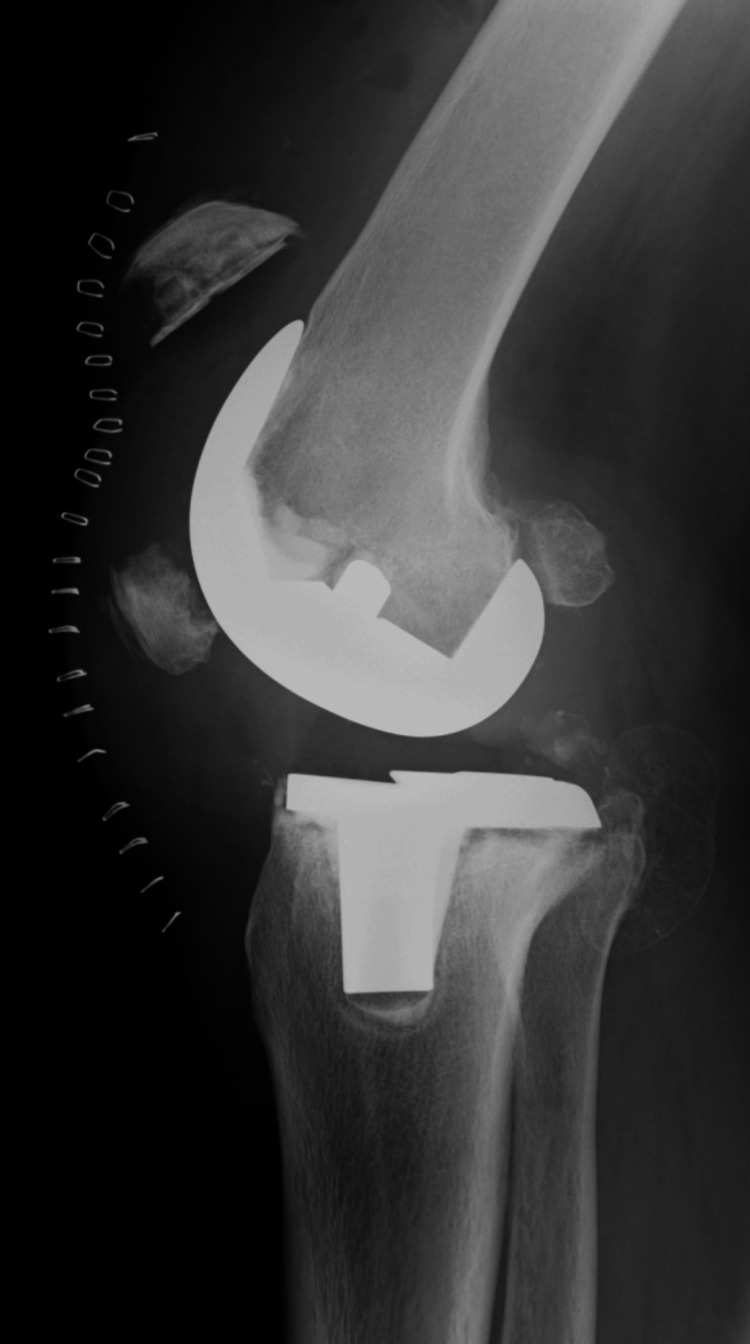
X-rays at the time of redisplacement of the fracture The X-ray revealed a redisplacement of the right patellar fracture.

**Figure 4:**
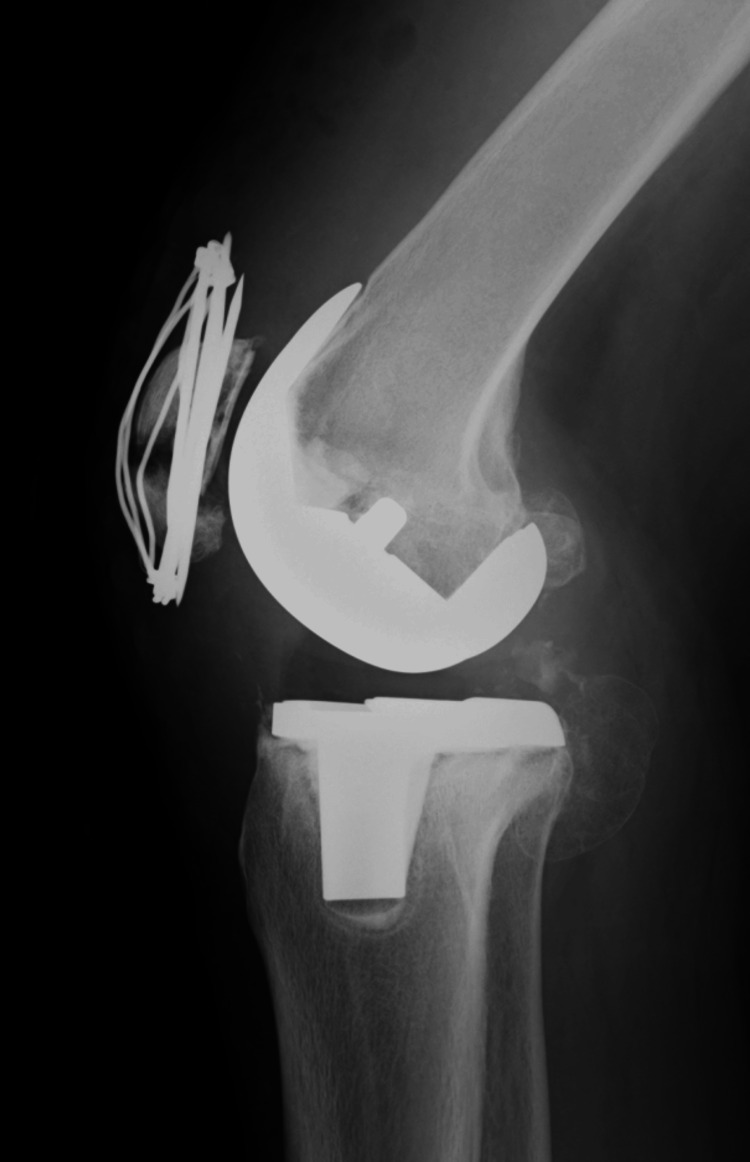
Postoperative X-ray following refracture surgery. At this time, revision osteosynthesis was performed using the AI wiring system (Aimedic MMT, Tokyo, Japan). Again, no loosening of the patellar component was observed during the operation and postoperative X-ray.

Osteosynthesis was performed using a polyethylene cable (NESPLON® Cable System; Alfresa Pharma Corporation, Osaka, Japan) for tension band wiring. No loosening of the patellar component was observed during the operation (Figure 2). On the sixth postoperative day, she dropped her cell phone on the floor and crawled on the floor looking for it, which caused right knee pain, and then X-ray showed a right patellar re-displacement of the fracture (Figure 3). Revision osteosynthesis was performed using the ring pins (AI-Wiring System; Aimedic MMT, Tokyo, Japan) for circumferential wiring. This time, there was no loosening of the patellar component during the operation (Figure 4).

**Figure 5:**
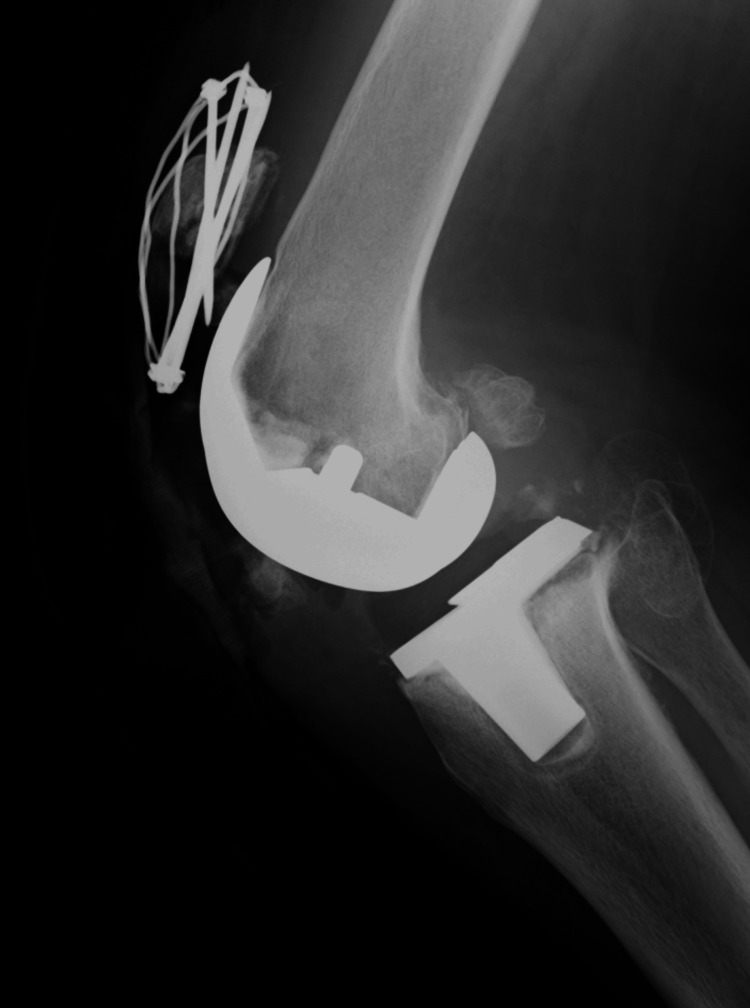
X-ray before implant removal Due to the inability to control the infection with debridement, bone union was not achieved, resulting in implant loosening.

**Figure 6:**
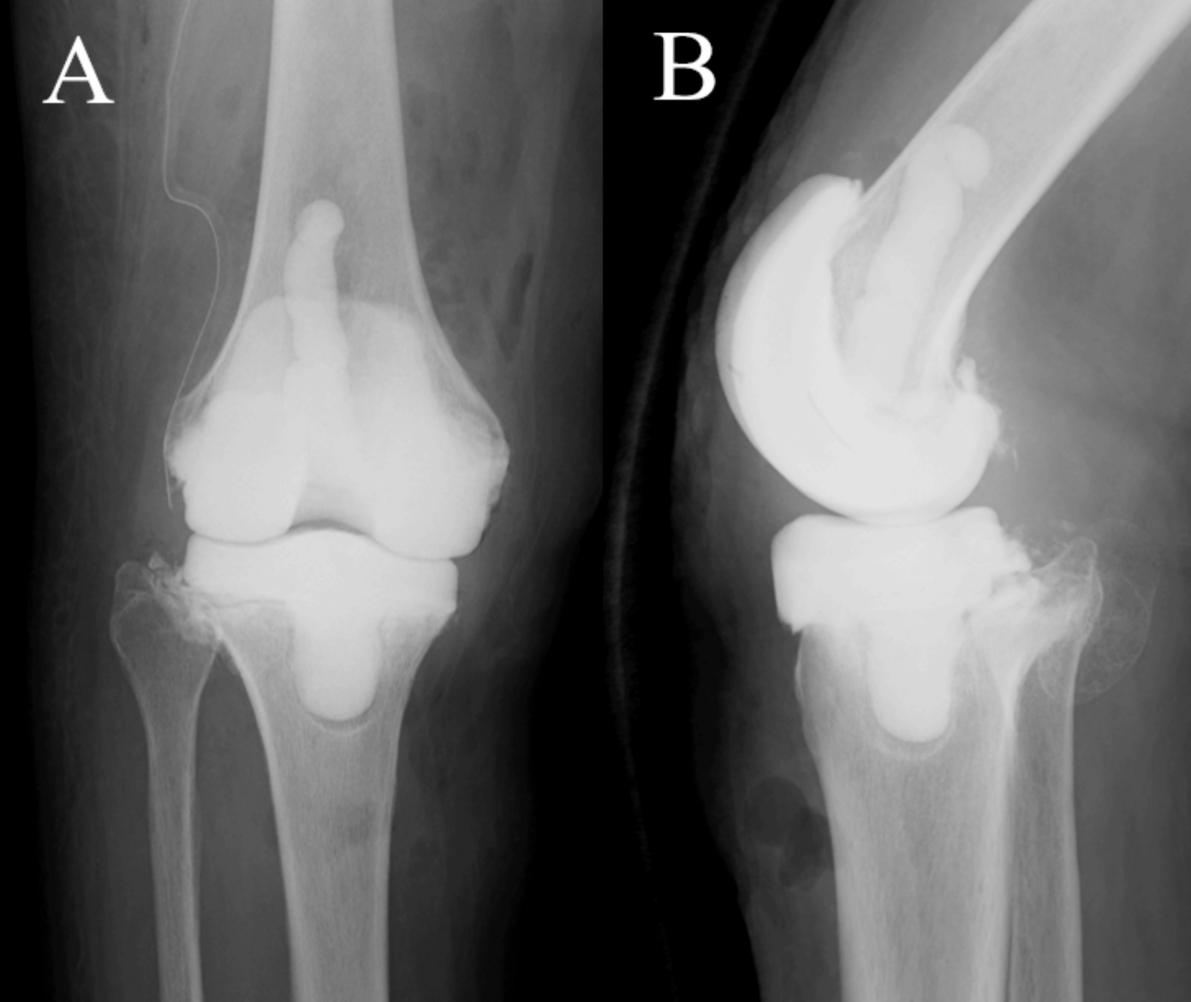
After antibiotic-loaded cement spacer replacement Uncontrolled infection led to removal of the implant and antibiotic-loaded cement spacer replacement, which was ineffective.  (A) Anteroposterior radiograph of the knee joint. (B) Lateral radiograph of the knee joint.

Postoperatively, a wound infection occurred. Due to the inability to control the infection with debridement of soft tissue, component loosening of the femur and tibia appeared, and the patella failed to achieve bone union, resulting in complete failure of the knee extension mechanism (Figure 5), which necessitated its removal, and 43 days after the primary fracture surgery, the patient underwent implant removal and its replacement with antibiotic-loaded cement spacers and patellectomy (Figure 6).

Although *Staphylococcus *species are often considered the causative bacteria [4,5], in this case, culture revealed *Enterobacter cloacae*. Three weeks after surgery, the wound reopened, the infection worsened, and profuse wound drainage resulted in systemic cutaneous candidiasis. Arthrodesis was deemed necessary due to the persistence of infection, the loss of soft tissue around the skin incision site, and the loss of the extensor mechanism following patellectomy. Since intramedullary nails are not commercially implemented in Japan, we decided to use a locking plate.

**Figure 7:**
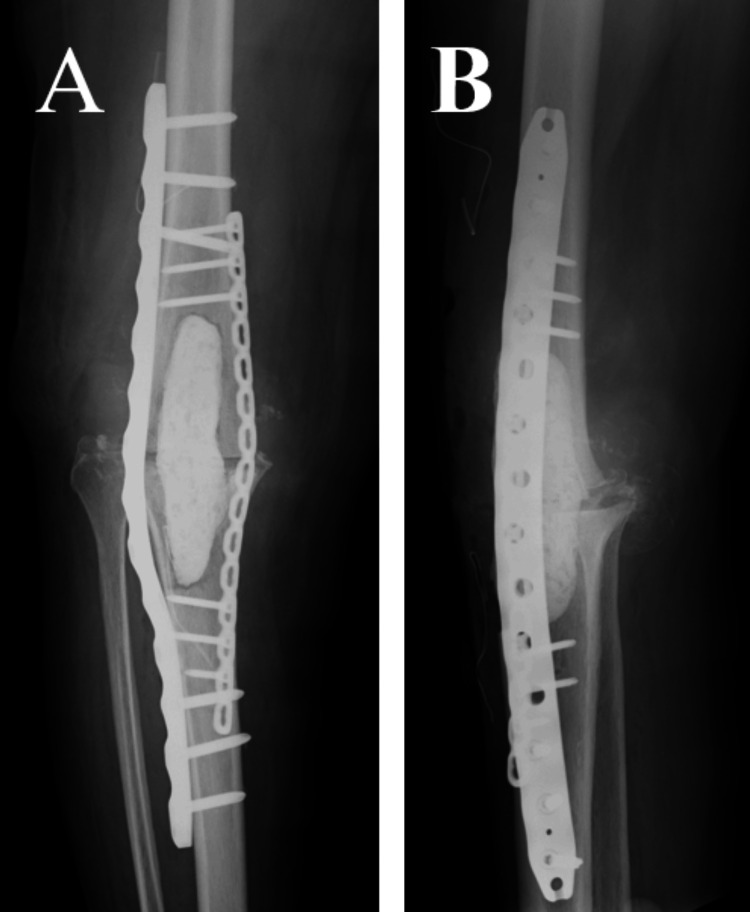
First-stage postoperative X-ray (A) Anteroposterior radiograph of the knee joint. (B) Lateral radiograph of the knee joint.

Arthrodesis was performed using a method based on the Masquelet technique [3]. In the first stage, thorough debridement and definitive fixation were performed. The cement inserted at the previous surgery was removed, then the intra-articular and intramedullary areas were scraped and cleaned. Both the medial and lateral condyles of the femur and tibia were flattened using a bone saw to fit the plates. The anterior cortex was fenestrated to allow for cement insertion. A locking plate (NCB Curved Femur Shaft Plate, 14 holes; Zimmer Biomet, Warsaw, USA) was placed on the lateral side, and a locking plate (Universal Locking System 3.5 mm Locking Reconstruction Plate Straight, 15 holes; Zimmer Biomet) was placed on the medial side. The plates were bent to fit the shape of the bone, with the lateral plate bent at the 7th hole from the proximal end and the medial plate bent between the 7th and 8th holes from the proximal end. A block of 40 g of cement mixed with 2 g of vancomycin** **and 80 mg of gentamicin** **was inserted into the defect (Figure 7).

**Figure 8:**
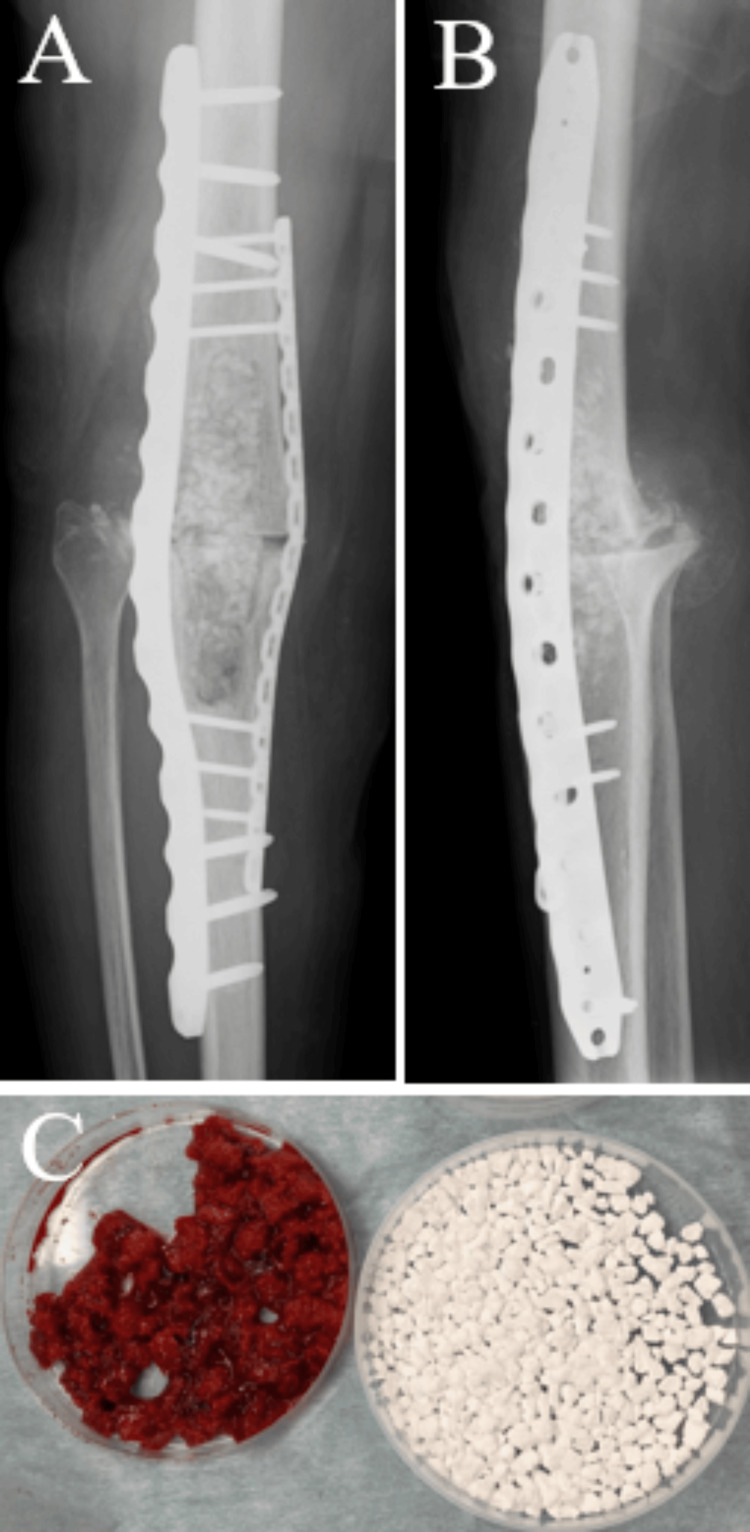
At the second stage, bone grafting was performed. (A) Anteroposterior radiograph of the knee joint. (B) Lateral radiograph of the knee joint. (C) Grafted with the same amount of beta-tricalcium phosphate (β-TCP) mixed with cancellous bone taken from the iliac bone.

Although a preoperative defect in the skin and soft tissue was present, complete wound closure was managed in the current surgical procedure. Six weeks after the first stage, as the secondary stage, bone grafting was performed. The cement was removed and grafted with the same amount of beta-tricalcium phosphate (β-TCP)mixed with cancellous bone taken from the iliac bone (Figure 8).

**Figure 9:**
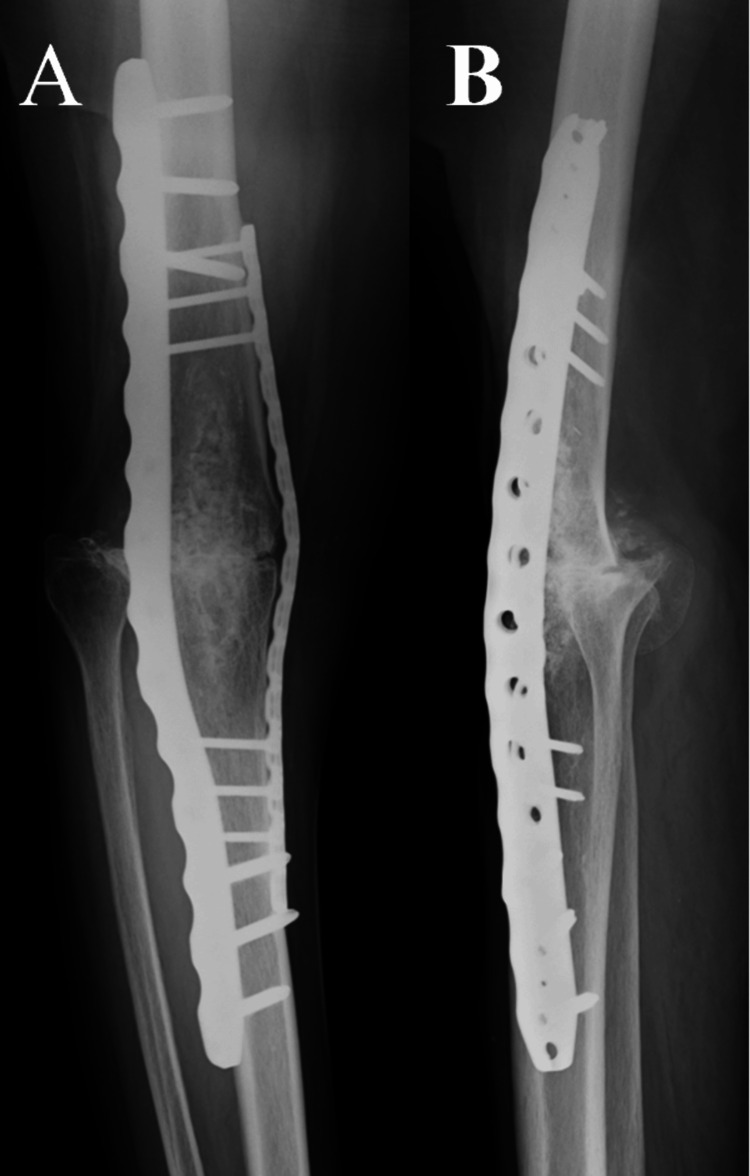
X-ray at 26 months postoperative (A) Anteroposterior radiograph of the knee joint. (B) Lateral radiograph of the knee joint.

The surgical site healed without complications within two weeks postoperatively. Intravenous antibacterial drugs were administered for 3 weeks after surgery, and oral antibacterial drugs were continued for 6 months after surgery. Bone union was observed at 12 months postoperatively. Due to a 5 cm limb shortening, a shoe lift was fabricated; however, she was unable to comprehend its use and ultimately did not utilize it. At 26 months postoperatively, she is capable of ambulating short distances but primarily relies on a wheelchair for mobility (Figure 9).

## Discussion

Patellar fractures following TKA are rare but challenging complications that can lead to significant morbidity, particularly when complicated by infection. These fractures have been reported to be associated with a notably high postoperative infection rate, ranging from 6.25% to 30.7% [6,7]. Therefore, in cases where the knee extension mechanism remains intact, conservative therapy can be considered a feasible treatment option. In this case, a series of complications, including re-displacement of the patellar fracture, deep infection, implant loosening, persistent soft tissue defects, and complete failure of the knee extension mechanism, necessitated a stepwise surgical approach culminating in knee arthrodesis. To address the infection and promote bone regeneration, the Masquelet technique was employed as the primary strategy.

The Masquelet technique is mainly used for the treatment of infected nonunion [5,8]. In the first stage, debridement of the infected tissue is performed, and the resulting bone defect is filled with an antibiotic-loaded bone cement spacer, followed by firm internal fixation with an intramedullary nail or locking plate. After 4 to 8 weeks, in the second stage, the cement is removed and bone grafting is performed. This method promotes rapid bone regeneration in the bone defect area. This technique has gained much attention in recent years because it addresses the limitations of external skeletal fixation, such as ADL disability due to large external devices and infections at the pin and wire insertion sites. Additionally, it offers the advantage of allowing range-of-motion exercises immediately after surgery without the need to worry about the timing of external fixation removal, which is a major concern for many patients.

In Europe and the United States, intramedullary nails have been reported to have advantages such as superior function and a high bone union rate for arthrodesis after failed TKA [3,9,10]. There are reports indicating that the use of intramedullary nails results in the formation of a membrane between the intramedullary nail and the cement spacer, thereby reducing the amount of bone graft required [11]. However, these types of implants are not commercially implemented in Japan, and locking plates and external skeletal fixation are often used. There are reports that a solid fusion rate for plate fixations is significantly lower compared to intramedullary nails and external fixations [3]. In our case, due to the history of mental disorder, external fixation could not be used, and plate fixation had to be chosen. However, since the success rate of conventional plate fixation is low, we tried applying the Masquelet technique for the treatment of infected nonunion. In the systematic review conducted by Morelli et al., the Masquelet technique for treating infected nonunion demonstrated an infection healing rate of 89.7% and a bone union rate of 91.1%, indicating excellent treatment outcomes [12].

In our case, arthrodesis was performed according to the Masquelet technique with definitive internal fixation using locking plates and bone cement filling in the primary stage. Autogenous cancellous bone and β-TCP granules were grafted into the bone defect in the secondary stage [5], six weeks after the primary stage. Her postoperative course was good, and this method was considered useful for arthrodesis in post-TKA infection, especially in countries where the use of intramedullary nails is not possible.

## Conclusions

We experienced a case of patellar fracture following TKA. Although osteosynthesis was performed, infection control was not achieved, and the subsequent patellectomy resulted in extensor mechanism failure, necessitating knee joint arthrodesis. The Masquelet technique, typically used for infected nonunion, was applied in this case, leading to a favorable outcome. Based on this experience, we consider the Masquelet technique to be a viable option for knee joint arthrodesis in cases where infection control following TKA has failed and the extensor mechanism has been compromised.
